# Redox-sensitive transient receptor potential channels in oxygen sensing and adaptation

**DOI:** 10.1007/s00424-015-1716-2

**Published:** 2015-07-07

**Authors:** Yasuo Mori, Nobuaki Takahashi, Onur Kerem Polat, Tatsuki Kurokawa, Norihiko Takeda, Masahiro Inoue

**Affiliations:** Laboratory of Molecular Biology, Department of Synthetic Chemistry and Biological Chemistry, Graduate School of Engineering, Kyoto University, Nishikyo-ku, Kyoto, 615-8510 Japan; Laboratory of Environmental Systems Biology, Department of Technology and Ecology, Hall of Global Environmental Studies, Kyoto University, Nishikyo-ku, Kyoto, 615-8510 Japan; Department of Cell Biology, Harvard Medical School, Boston, MA 02115 USA; Department of Cardiovascular Medicine, Graduate School of Medicine, The University of Tokyo, Bunkyo-ku, Tokyo, 113-8655 Japan; Department of Biochemistry, Osaka Medical Center for Cancer and Cardiovascular Diseases, Higashinari-ku, Osaka, 537-8511 Japan

**Keywords:** TRP channels, Oxygen, Hypoxia, Vagus, Carotid body

## Abstract

Regulation of ion channels is central to the mechanisms that underlie immediate acute physiological responses to changes in the availability of molecular oxygen (O_2_). A group of cation-permeable channels that are formed by *transient receptor potential* (TRP) proteins have been characterized as exquisite sensors of redox reactive species and as efficient actuators of electric/ionic signals in vivo. In this review, we first discuss how redox-sensitive TRP channels such as TRPA1 have recently emerged as sensors of the relatively inert oxidant O_2_. With regard to the physiological significance of O_2_ sensor TRP channels, vagal TRPA1 channels are mainly discussed with respect to their role in respiratory regulation in comparison with canonical pathways in glomus cells of the carotid body, which is a well-established O_2_-sensing organ. TRPM7 channels are discussed regarding hypoxia-sensing function in ischemic cell death. Also, ubiquitous expression of TRPA1 and TRPM7 together with their physiological relevance in the body is examined. Finally, based upon these studies on TRP channels, we propose a hypothesis of “O_2_ remodeling.” The hypothesis is that cells detect deviation of O_2_ availability from appropriate levels via sensors and adjust local O_2_ environments in vivo by controlling supply and consumption of O_2_ via pathways comprising cellular signals and transcription factors downstream of sensors, which consequently optimize physiological functions. This new insight into O_2_ adaptation through ion channels, particularly TRPs, may foster a paradigm shift in our understanding in the biological significance of O_2_.

## Introduction

Molecular oxygen (O_2_) is an essential substrate for life, because of its role in the generation of adenosine triphosphate (ATP) which is a major source of energy in aerobic organisms. It is therefore fundamental that aerobic organisms sense and respond to hypoxia (low O_2_ environments), thus allowing them to adapt to variable habitats and physiological situations. Physiological responses to hypoxia can be classified into immediate acute (~s) and later (subacute to chronic) forms (~m to h). Later responses depend at least in part on hypoxia-inducible transcription factors (HIFs) [91, 112], which determine the expression of numerous gene-encoding enzymes, transporters, and growth factors. Immediate acute responses rely mainly on adaptive changes mediated by O_2_-regulated ion channels, which regulate cell excitability, contractility, and secretory activity. Respiratory and cardiovascular systems can adjust themselves rapidly to maintain O_2_ delivery to the most critical organs, such as the brain and heart. As early as 1868, Pflüger recognized that hypoxia stimulates respiration [79], which spurred a search for O_2_-sensitive receptors within the brain and at various sites in the peripheral circulatory system. Jean-Francois Heymans and his son, Corneille Heymans, reported that stimulation of breathing by hypoxia is a reflex triggered by the carotid bodies (CBs) located at the bifurcation of the common carotid arteries [37]. Chemosensory inputs from CBs are carried within the glossopharyngeal nerves toward the medullary centers, which generate stimuli to change the respiratory pattern. With regard to the mechanisms underlying hypoxia-sensing in the CBs, it is generally accepted that hypoxia inhibits K^+^ channels to depolarize chemoreceptor glomus cells, leading to the activation of voltage-dependent Ca^2+^ channels and exocytosis [115]. However, the exact channel subtypes and direct mediators responsible for hypoxia-sensing remain controversial [61].

The physiological significance and hypoxia-sensing mechanisms of the non-CB chemoreceptors remain unclear and represents an important area that requires much further research. Recently, a major advance in our understanding of the function of non-CB chemoreceptors came with the identification of *transient receptor potential* (TRP) cation-permeable channels, which have exquisite sensitivity to redox reactive species. Among this group of TRP channels, the TRPA1 channel has emerged as a sensor in non-CB chemoreceptors to detect deviation of O_2_ availability (hypoxia and hyperoxia) from normoxia in vivo [81, 101]. Given that TRPA1 channels are predominantly expressed in vagal and sensory neurons, the responses to mild hypoxia are attributable mainly to vagal nerves themselves or lung airway neuroepithelial bodies (NEBs) and aortic bodies (ABs) innervated by vagal nerves [115]. These findings further suggest that there are different O_2_-signaling mechanisms that respond to varying degrees of hypoxic stimulus. Thus, studies on the redox-sensitive TRP channels opened up a new avenue for studying O_2_-sensing organs and the O_2_ environment that is formed within our body.

### What are redox-sensitive TRP channels?

The cellular redox status depends on a balance between the levels of intracellular antioxidants and redox reactive species, including reactive oxygen and nitrogen species and other electrophilic molecules. It was generally understood that the disruption of cellular redox homeostasis by excessive production of redox reactive species leads to oxidative damage to membrane lipids, proteins, and DNA [15]. However, in the past two decades, several lines of evidence have suggested that redox reactive species also serve as signaling molecules that regulate biological and physiological processes [26].

One particular group of TRP channels function as exquisite sensors of redox reactive species and as efficient actuators of electric and ionic signal in vivo [52]. The TRPM2 channel, the first identified redox-sensitive TRP channel, is activated indirectly by H_2_O_2_ through the production of nicotinamide adenine dinucleotide and its metabolites, ADP-ribose and cyclic ADP-ribose [35, 78]. Accumulated evidence indicates that TRPM2 mediates H_2_O_2_-activated Ca^2+^ influx that mediates cell death [35] and irradiation-activated Ca^2+^ influx that causes irreversible loss of salivary gland function [59]. TRPM2 also mediates H_2_O_2_-activated Ca^2+^ or cation influx that drives insulin secretion in pancreatic β-cells [104, 107]. Furthermore, studies using *Trpm2* gene knockout (KO) mice have revealed that H_2_O_2_-activated Ca^2+^ influx through TRPM2 contributes to innate immune responses via chemokine production in monocytes [119], neutrophil adhesion during myocardial ischemia/reperfusion injury [39], and NLRP3 inflammasome activation in macrophages [122].

In addition to the indirect redox-sensing mechanism that involves TPRM2, direct sensing through cysteine (Cys) modification has emerged as a prominent mechanism underlying activation of various TRP channels [103]. Oxidative modifications of Cys residues by H_2_O_2_, nitric oxide (NO), and reactive disulfides have been demonstrated for TRPC5 [120], which was originally identified from the mouse brain as a receptor activated Ca^2+^-permeable cation channel linked to phospholipase Cs [74, 80]. NO and reactive disulfides directly modify Cys residues (Cys553 and Cys558) located on the N-terminal side of the pore-forming region between S5 and S6 transmembrane helices via S-nitrosylation and disulfide exchange reactions, respectively, in mouse TRPC5. In vascular endothelial cells, TRPC5 activation induced by NO via nitrosylation enhances Ca^2+^ influx, which induces NO production by endothelial type NO synthase (eNOS) [120]. This raises the possibility that TRPC5 mediates a positive feedback loop of NO production upon vasodilator stimulation in vascular endothelial cells [28, 120]. Interestingly, TRPC5 is also activated by the reducing agent dithiothreitol and extracellular-reduced thioredoxin [118]. The closest relatives of TRPC5 are TRPC1 and TRPC4, as well as thermosensor channels TRPV1, TRPV3, and TRPV4, which carry Cys residues corresponding to Cys553 and Cys558 on TRPC5 protein [120]. Indeed, these channels are targets of nitrosylation that leads to channel activation. TRPV1 also shows sensitivity to phenylarsine oxide and allicin from garlic through covalent modification of Cys residues located in the C-terminal and N-terminal regions [12, 87].

More recently, the TRPA1 channel has been shown to open upon oxidative Cys modification by pungent compounds and inflammatory mediators [38, 62, 102]. Originally identified TRPA1 activators are pungent natural compounds that include cinnamaldehyde, allyl isothiocyanate, and α,β-unsaturated aldehydes from plants such as mustard, onion, cinnamon, and wasabi, and the pungent garlic compound allicin (these compounds are potentially susceptible to the nucleophilic attack at the sulfhydryl group of Cys residues), cold temperature, receptor stimulation, and cannabinoids [5, 7, 47, 63, 97]. Later examinations of various noxious compounds finally led to the understanding that electrophilic pungent compounds that covalently modify Cys residues through mechanisms such as Michael addition, are commonly potent activators of TRPA1 channels [38, 62].

**Fig. 1:**
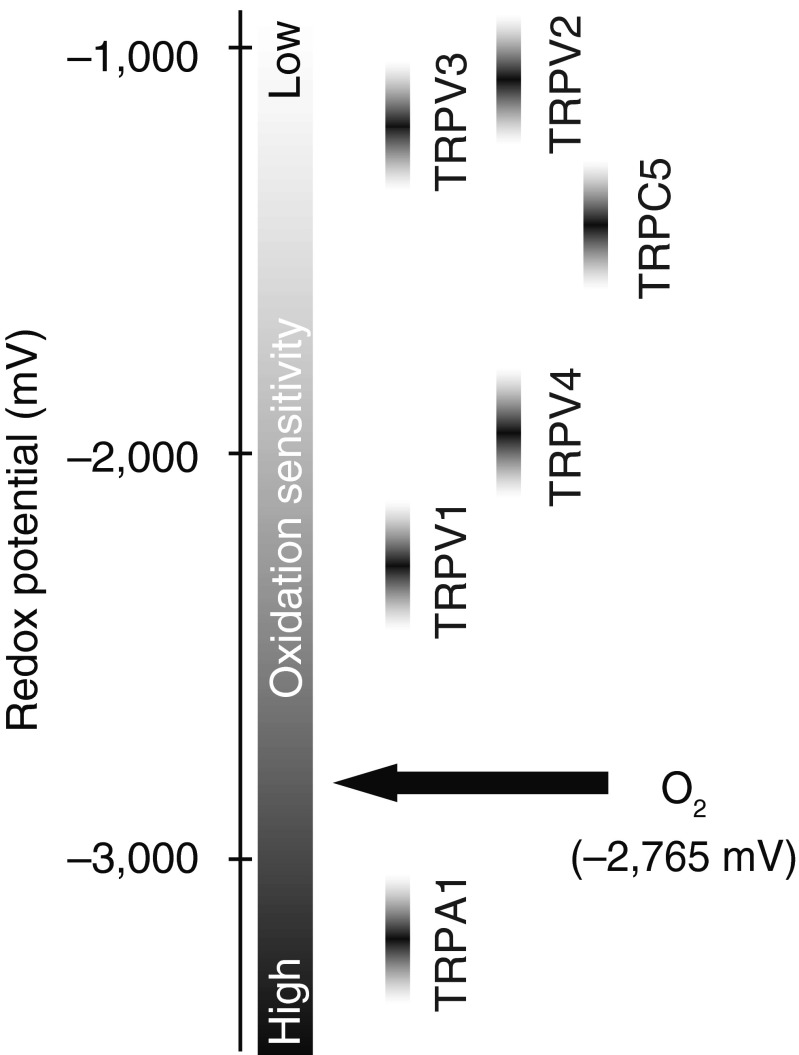
Threshold redox potentials for activation of redox-sensitive TRP channels

Considering the distinct redox reactivity of each oxidizing chemical species, particular redox sensitivity of TRP channels should be quantified in terms of sensitivity to these species. This was attained through systematic comparison of the responses of redox-sensitive TRP channels with a congeneric series of reactive disulfides, which show different electron acceptor (oxidation) abilities indicated as redox potentials that are obtained using rotating disc electrode voltammetry [101]. TRP channel activity was correlated with redox potentials of reactive disulfide stimuli, revealing threshold redox potentials for respective TRPs (Fig. 1). Strikingly, among the TRPs tested, only TRPA1 responded to inert oxidants/electrophiles with a redox potential of −2950 mV. The redox potential of O_2_ (−2765 mV) is less negative than the threshold redox potential for TRPA1 (approximately −3400 mV) but is more negative than these for the other channels investigated, suggesting that TRPA1 is activated by O_2_ (a weak oxidant) to function as a hyperoxia sensor. Indeed, only TRPA1 responded to hyperoxic solutions prepared by bubbling with O_2_ gas in a concentration-dependent manner [101]. Thus, among TRP channels, TRPA1 has the highest oxidation sensitivity, which enables TRPA1 to respond to an inert oxidant such as O_2_.

### TRPA1 as an O_2_ sensor

In higher animals, particularly mammals, the respiratory and cardiovascular systems must rapidly adjust themselves to maintain O_2_ delivery to the most critical organs, such as the brain and heart. In mammals, it is understood that the CBs detect changes in partial O_2_ pressure (PO_2_) through K^+^ channel activities in arterial blood [30, 71, 115]. Sensory and vagal afferent neurons, which project nerve endings throughout the body, have also been proposed to detect hypoxia in organs, such as the airway, lungs, and heart, under ischemia and other conditions of low O_2_ supply [17, 32, 41, 60]. However, the characteristics and mechanisms of hypoxia detection by non-CB chemoreceptors including sensory and vagal neurons, have yet to be fully defined [60]. Recently, a major advance in our understanding of the function of non-CB chemoreceptors came with the demonstration that the TRPA1 channel, which is expressed in non-CB chemoreceptors is capable of detecting changes in O_2_ availability in vivo [81, 101].

As described above, systematic evaluation of TRP channels using reactive disulfides with different redox potentials led to our finding that TRPA1 can sense O_2_ [101]. Notably, Cys oxidation is not the only mechanism that underlies O_2_ sensing in TRPA1 channels. Indeed, hypoxic solutions prepared by bubbling with N_2_ gas induce robust TRPA1 responses; TRPA1 activation shows an inverted bell-shaped O_2_-dependence curve with a minimum at PO_2_ of 137 mmHg (18 %), which is slightly below the atmospheric PO_2_ of 152 mmHg (20 %).

**Fig. 2:**
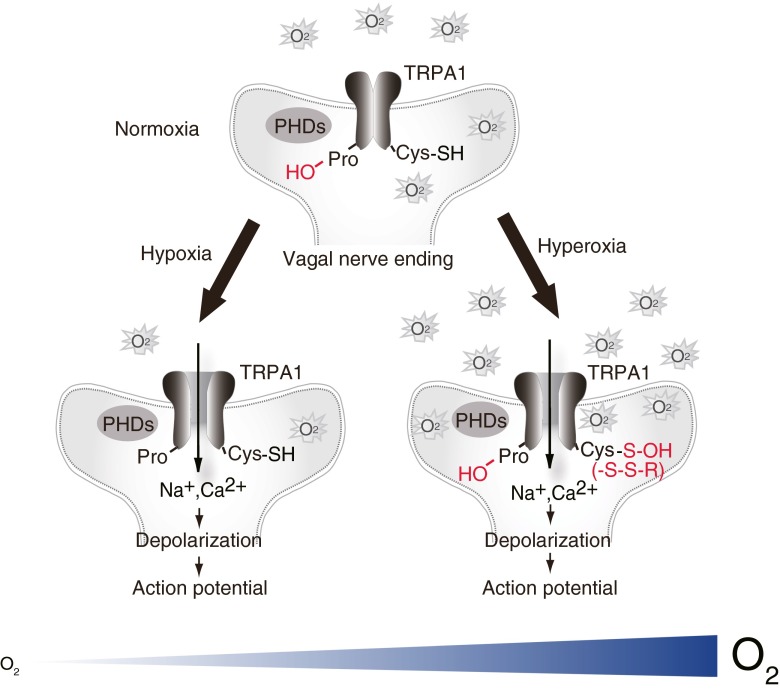
Model for TRPA1-mediated O_2_-sensing mechanisms at the vagal nerve ending. PHDs hydroxylate conserved Pro394 within the N terminus ankyrin repeat of TRPA1 during normoxia. A decrease in O_2_ concentrations diminishes PHD activity and relieves TRPA1 from inhibition, leading to its activation in hypoxia. O_2_ during hyperoxia oxidizes Cys633 (and possibly Cys856), thereby activating TRPA1. This Cys oxidation may dominate the inhibition by Pro hydroxylation to activate TRPA1

O_2_ sensing by TRPA1 is based upon disparate processes, such as proline (Pro) hydroxylation by Pro hydroxylases (PHDs) and direct oxidation of Cys residues [101] (Fig. 2). During normoxia, PHDs hydroxylate conserved Pro394 within the 10th ankyrin repeat domain of human TRPA1 to inhibit its activity. During hypoxia, the decrease in O_2_ concentration diminishes PHD activity, relieving TRPA1 from the inhibitory action of Pro hydroxylation to lead to its activation. This recovery of TRPA1 activity is likely dependent on the insertion of fresh, unmodified TRPA1 proteins into the plasma membrane or an unidentified dehydroxylation of modified proteins through an unidentified molecular mechanism. During hyperoxia, O_2_ activates TPRA1 by oxidizing Cys633, Cys856, or both. Cys633 and Cys856 are located within the 17th ankyrin repeat domain and the intracellular linker region between S4 and S5, respectively, in human TRPA1. TRPA1 can take at least two oxidized forms during hyperoxia: a relatively unstable oxidized state (state 1) readily reversed by glutathione and a relatively stable oxidized state (state 2). Sulfhydryl groups on the key Cys residues (Cys633 and Cys856) may be modified to sulfenic acid (S-OH) in state 1 and form disulfide bonds (S-S) in state 2. This oxidation mechanism overrides the inhibition by Pro hydroxylation to activate TRPA1.

In mice, exposure to hyperoxic (100 % O_2_) or hypoxic (10, 13, and 15 % O_2_) gas via a tracheal cannula significantly enhances discharges of afferents in the cervical vagal trunk and in the superior laryngeal vagal branch innervating the mucosa of the larynx, as shown by a multifiber neurogram. However, disruption of the *Trpa1* gene abolishes the enhancement of nerve discharges by hyperoxia and mild hypoxia (15 % O_2_) and delays that by severe hypoxia (10 and 13 % O_2_) [101]. Notably, TRPA1 antagonism abolishes the respiratory responses to mild hypoxia (13 % O_2_) but not to severe hypoxia (7 % O_2_) in conscious mice [81]. These findings raise possibility that there are different O_2_ signaling mechanisms that respond to varying degrees of hypoxic stimulus. In mild hypoxia, the respiratory responses appear to be crucially dependent on TRPA1 channels, as TRPA1 antagonism abolishes the response. Given that TRPA1 channels are predominantly expressed in vagal and sensory neurons [68], it is possible that the responses to mild hypoxia are attributable mainly to non-CB chemoreceptors including vagal nerves, NEBs, and/or ABs in mice. Conversely, during severe hypoxia, the respiratory responses may be more dependent on hypoxia-sensitive K^+^ channels in the CBs, with little involvement of the TRPA1 system, in agreement with studies using *Trpa1*-deficient mice [101]. The finding of O_2_ sensitivities of TRPA1 underscores the importance of non-CB chemosensitive mechanisms in hypoxic respiratory responses in mammals.

### TRPM7 as another O_2_ sensor candidate among TRP channels

TRPM7 is an important candidate O_2_ sensor. This TRP channel is characterized by its unique “chanzyme” structure comprising the kinase domain as well as the transmembrane ion channel pore permeable to cations such as Mg^2+^, Ni^2+^, Zn^2+^, and other trace metals [65, 67, 84]. Tymianski’s group originally demonstrated activation of TRPM7 by anoxic condition using cultured neurons subjected to oxygen-glucose deprivation [1]. ROS and RNS have been suggested to mediate this mode of TRPM7 activation. In our systematic evaluation of different redox-sensitive TRP channels, we also observed that TRPM7-like TRPA1 is activated by application of hypoxic solution prepared by bubbling N_2_ gas [101]. Anoxia/hypoxia-induced activation of TRPM7 plays an important role in non-excitotoxic ischemic brain injury [99], in which large reductions in extracellular divalents, acidosis, and oxidative stress are induced [58, 94, 95]. All these conditions potentiate TRPM7 activity, although TRPM7 conducts only a few pA of inward currents under physiological pH levels, extracellular Ca^2+^ and Mg^2+^ concentrations, and low oxidative stress [53, 67, 84, 114]. The C-terminal kinase domain excised from the channel domain has been implicated in the cell death process [18, 54].

Wide expression of TRPM7 suggests its general biological importance shared by different types of cells [67, 84]. After it was reported that disruption of TRPM7 in DT-40 B cell lines affect their survival [67, 90], evidence has been accumulating for the involvement of TRPM7 in proliferation and metastasis of various forms of cancer cells [33, 34, 45, 123]. TRPM7 also regulates a variety of basic cellular responses, such as cell adhesion [13, 72], polarization [89], migration [13, 113], and volume regulation [73]. Moreover, TRPM7 is essential for embryonic development before day 7.5 of embryogenesis and for T cell growth needed for thymopoiesis [46]. In regulating these cellular responses, particularly in proliferation, Mg^2+^ permeation that controls cellular Mg^2+^ homeostasis and downstream phosphoinositide 3-kinase is likely an important function of TRPM7 channels [67, 86, 90]. Thus, assuming that hypoxia-induced activation is the common feature shared by TRPM7 channels in different tissues and cell types, it is possible that decreases in local O_2_ levels in vivo by changes in body architecture, during development, and changes in climate, can modulate TRPM7 function to modify ionic homeostasis and/or downstream signaling cascades.

Activation of TRPC6 by hypoxia [116] and its underlying mechanism, which may contribute to extension of the concept above, will be discussed elsewhere in this Special Issue.

### Ubiquitous expression of O_2_ sensor TRP channels (TRPA1 and TRPM7)

TRPA1, originally named p120, was first cloned from fibroblasts by Jaquemar and colleagues when a novel mRNA was discovered in fibroblasts but was completely absent in SV40-transformed cells and mesenchymal tumor cell lines [44]. The most interesting feature of TRPA1 with many ankyrin repeats (ranging from 15 to 18 repeats) was intriguing to the investigators at that time owing to the fact that the only known similarity in structure belonged to an insect toxin called latrotoxin. Although the structure conformed to the general structure of TRP channels, phylogenetic analysis revealed it to be distant from the currently known TRPs, thus prompting it to be placed as a separate subfamily [44]. The group also observed that the *TRPA1* gene expression was relatively low and difficult to detect with northern blot analysis and required more sensitive polymerase chain reaction (PCR) technology. Despite this, TRPA1 was detected in numerous tissues [44] and was confirmed later in subsequent studies (Tables 1 and 2).

**Table 1:** Expression of TRPA1 in neuronal cells and tissues, function, and method of detection, shown in chronological order

Expressed in cell and tissue	Function (including suggested function)	Species	Method of detection	Reference	Year
Dorsal root ganglion neurons	Noxious cold sensor, thermosensation	Rat, mouse	Northern blotting, in situ hybridization, calcium imaging, electrophysiology	Story et al. [97]	2003
Sympathetic superior cervical ganglion neurons	Sole cold sensor, thermosensation	Murine	Calcium imaging	Smith et al. [96]	2004
Trigeminal neurons (C-fibers)	Nociception, sensory	Rat	In situ hybridization, immunohistochemistry	Kobayashi et al. [51]	2005
Dental primary afferents	Thermosensation	Rat	Immunohistochemistry, single-cell RT-PCR, whole-cell recordings	Park et al. [77]	2006
Geniculate ganglion	Somatosensory or gustatory function, nociception, thermosensing	Rat	RT-PCR, in situ hybridization	Katsura et al. [49]	2006
Primary sensory neurons	Mechanosensory transduction, nociception	Rat	Quantitative PCR, immunofluorescence staining, cystometry	Du et al. [20]	2007
Lung afferent fibers	Respiratory, nociception	Mouse	Single-cell RT-PCR, whole-cell patch-clamp recordings	Nassenstein et al. [70]	2008
Masticatory muscle afferent fibers	Craniofacial muscle nociception, mechanical hyperalgesia	Rat	Immunohistochemistry, behavioral studies	Ro et al. [83]	2009
Trigeminal sensory afferents, spinal dorsal horn	Nociception	Rat	Electron microscopy, immunohistochemistry	Kim et al. [50]	2010
Nodose, jugular and petrosal ganglions	Putative somatic, chemo- and somato-sensation, somato and visceral sensation	Rat	In situ hybridization	Hondoh et al. [40]	2010
Inhibitory motorneurons of the intestine	Inhibition of spontaneous neurogenic contractions and transit of colon	Mouse	RT-PCR, immunofluorescence, calcium imaging	Poole et al. [82]	2011
Dura	Headache	Mouse	Immunohistochemistry	Huang et al. [42]	2012
Vestibular ganglia	Vestibular function, vertigo	Rat	RT-PCR, in situ hybridization, immunohistochemistry, calcium imaging	Kamakura et al. [48]	2013
Vagina epithelium, wall nerve fibers	Neurotransmission	Human	Immunohistochemistry, RT-PCR	Uckert et al. [108]	2015

**Table 2:** Expression of TRPA1 in non-neuronal cells and tissues, function, and method of detection, shown in chronological order

Expressed in cell and tissue	Function (including suggested function)	Species	Method of detection	Reference	Year
Hair cell of the ear	Hair cell transduction, mechanosensation (debated)	Zebrafish, mouse	In situ hybridization, siRNA	Corey et al. [14]	2004
Urethra	Tone of urethral preparations, afferent and efferent sensory signaling of the human outflow region	Human	Western blotting, immunohistochemistry, functional in vitro investigations	Gratzke et al. [31]	2009
Skin	Keratinocyte differentiation, inflammation	Human	Quantitative PCR, microarray	Atoyan et al. [4]	2009
Developing cochlea	Normal cochlear function	Mouse	Quantitative PCR	Asai et al. [3]	2010
Olfactory epithelium	Olfactory chemosensation, Olfactory adaptation, olfactory–trigeminal interaction, olfactory epithelium fluid homeostasis.	Mouse	Immunohistochemistry	Nakashimo et al. [69]	2010
Dental pulp fibroblasts	Thermosensation	Human	RT-PCR, western blotting, immunohistochemistry	Karim et al. [22]	2011
Lung fibroblasts and epithelial cells	Pathogenesis of airway diseases	Human	Calcium imaging	Mukhopadhyay et al. [66]	2011
Pancreatic beta cells	Insulin secretion	Rat	Immunohistochemistry, RT-PCR, western blotting, calcium imaging	Cao et al. [9]	2012
Astrocytes in the superficial laminae of trigeminal caudal nucleus	Inflammation	Rat	Immunoelectron microscopy	Lee et al. [56]	2012
Olfactory bulb	Olfactory transduction	Mouse	RT-PCR	Dong et al. [19]	2012
Pulmonary epithelial cells	Inflammation	Human, Porcine	Immunohistochemistry	Buch et al. [8]	2013
Peridontal ligament cells	Mechanoreception	Human	DNA microarray	Tsutsumi et al. [106]	2013
Odontoblasts	Sensing membrane stretching, low-temperature stimulation	Rat	Immunohistochemistry	Tsumura et al. [105]	2013
Digestive system, enteroendocrine cells	Secretion possibly to aid digestion	Mouse	In situ hybridization, Immunofluorescence staining	Cho et al. [11]	2014
Uvea	Thermosensation	Human	Quantitative PCR, calcium imaging	Mergler et al. [64]	2014

The function of TRPA1 became evident 4 years later where TRPA1 was shown to mediate sensation of noxious and painful cold and to be expressed in the dorsal root ganglion (DRG) neurons. TRPA1 co-localizes with TRPV1 (a heat-sensing TRP channel) expressing sensory neurons rather than TRPM8-positive sensory neurons, indicating separate cold-sensing modalities [97]. This was particularly interesting as TRPM8 is different from TRPA1 in responding to mild cold temperatures as well as to different sets of organic compounds [97]. Since its discovery, TRPA1 has been reported in most sensory neurons targeting vital organs (see the non-extensive Table 1 below for TRPA1 expression in neuronal populations and nociception) [20, 40, 42, 48–51, 70, 77, 82, 83, 96, 97, 108]. To date, TRPA1 has been also detected in non-neuronal cells such as hair cells of the ear, urethra, skin, olfactory epithelium, dental pulp, uvea, vagina, and pulmonary epithelial cells, and this list is still growing (Table 2) [3, 4, 8, 9, 11, 14, 19, 22, 31, 55, 56, 64, 66, 69, 105, 106]. In 2004, Corey and colleagues proposed the idea that, TRPA1 may be involved in mechanosensation in the hair cell epithelia [14]. A follow-up study nearly half a decade later performed by the same group showed later however that TRPA1 KO mice exhibited normal vestibular function, normal startle reaction following loud auditory stimuli and normal hearing [55].

TRPM7 was first cloned from the rat brain library. Ryazanova and colleagues investigated deletion of the TRPM7 kinase domain in mice [85]. They showed that homozygous mice with TRPM7 lacking the protein kinase domain (denoted as TRPM7^Δkinase^) were embryonically lethal, while TRPM7^Δkinase^ heterozygous mice showed impaired magnesium homeostasis. TRPM7^Δkinase^ heterozygous mice showed low magnesium concentration in the plasma, erythrocytes, and bones. Magnesium impairment was further demonstrated with data obtained from mice fed a poor magnesium diet. Mice with TRPM7^Δkinase^ showed clasping, tremor, and seizures consistent with impairment in magnesium homeostasis. To elucidate the complete functional profile of the TRPM channel family, Fonfria and colleagues analyzed TRPM7 temporal channel tissue distribution by quantitative PCR [27]. Their study revealed TRPM7 expression in the brain, pituitary, heart, lung, liver, fetal liver, skeletal muscle, stomach, intestine, spleen, macrophages, adipose, pancreas, prostate, placenta, cartilage, bone marrow, and bone. Highest expression was in the pituitary, heart, adipose, and bone, and lowest expression was in cartilage, liver, and bone marrow [27]. Subsequent studies employing various techniques with varying sensitivity confirmed the findings (Table 3) [1, 2, 6, 10, 16, 21, 23, 25, 27, 29, 36, 73, 84, 92, 110, 111, 114, 117, 121]. Thus, TRPA1 and TRPM7 have been shown to be ubiquitous in many tissues and cells. Since the function of these channels was shown to be tissue specific, the spatial and temporal expressions of these channels are important clues for the ever growing list of functions.

**Table 3:** Expression of TRPM7 in cells and tissues, function, and method of detection, shown in chronological order

Expressed in cell and tissue	Function (including suggested function)	Species	Method of detection	Reference	Year
Heart, brain, spleen, lung, liver, skeletal muscle and kidney	Calcium channel, serine-threonine kinase	Mouse	Electrophysiology, nothern blotting	Runnels et al. [84]	2001
Cortical neurons	Magnesium homeostasis, excitotoxicity	Mouse	Electrophysiology, radioisotope techniques	Aarts et al. [1]	2003
Vascular smooth muscle cells	Mg^2+^ homeostasis	Rat, Mouse, Human	Biochemical, genetical and pharmacological tools	He et al. [36]	2005
Liver (hepatocytes)	Cell proliferation	Zebrafish, human	RT-PCR, immunocytochemistry, patch-clamp recordings, calcium imaging	Boustany et al. [21], and Elizondo et al. [23]	2008, 2005
Heart, pituitary, bone, adipose tissue	ND	Human	RT-PCR	Fonfria et al. [27]	2006
Epithelial cells	Stretch- and swell-sensitive ion channel, cell volume regulation	Human	Single channel recordings, RT-PCR	Numata et al. [73]	2007
Prostate	ND	Rat	RT-PCR	Wang et al. [111]	2007
Human lung mast cells (HLMCs), human mast cell lines (LAD2 and HMC-1)	Release of proinflammatory mediators, cell survival	Human	Electrophysiology, RT-PCR	Wykes et al. [117]	2007
Hippocampal neurons (CA1 neurons)	Excitotoxicity, Ca^2+^ paradox	Mouse	Electrophysiology	Wei et al. [114]	2007
Rumen epithelial cells	Magnesium transport pathways	Ovine	RT- PCR, western blotting, flow cytometry, immunocytochemistry, magnesium imaging	Schweigel et al. [92]	2008
Human osteoblast-like cells (MG-63, SaOS and U2-OS cells)	Cell proliferation	Human	Cell proliferation, PCR, calcium and magnesium imaging	Abed et al. [2]	2009
Bone-marrow derived mesenchymal stem cells	Cell survival	Mouse	RT-PCR, immunocytochemistry, electrophysiology	Cheng et al. [10]	2010
Urothelial cells	Polymodal sensing	Mouse	RT-PCR, immunocytochemistry, patch-clamp recordings, calcium imaging	Everaerts et al. [25]	2010
Retina (cone outer segments)	Magnesium homeostasis	Mouse	RT-PCR, northern blotting, in situ hybridization	Gilliam and Wendsel [29]	2011
Atrial myocytes	Fibrogenesis	Human	Whole-cell patch-clamp recordings, RT-PCR, western blotting	Zhang et al. [121]	2012
Trigeminal neurons, dorsal root ganglion neurons	Cell proliferation, organ development, Mg^2+^ homeostasis	Mouse	Quantitative PCR	Vandewauw et al. [110]	2013
Endometrial stromal cells	Cell proliferation	Human	Quantitative PCR, Immunocytochemistry, calcium imaging, whole-cell patch-clamp recordings	De Clercq et al. [16]	2015

*ND* not determined

### What is the significance of the ubiquity of O_2_ sensor TRP channels in the body?

It is important to address the primary significance of O_2_-sensing TRP channels that are ubiquitously expressed in the body. We suggest that these O_2_ sensors play key roles in the molecular mechanisms which underlie the O_2_-sensing ability of chemoreceptor (or chemoreceptor-like) cells localized ubiquitously in a variety of tissues and organs. It is possible that TRP O_2_ sensors detect local O_2_ availability and contribute to fine tuning of local O_2_ levels, which cannot be done by the CB alone, in the respective organs and tissues and in their subareas. Information of detected local O_2_ availability (partial pressure) may be transmitted through neurons, as discussed above and/or humoral factors to control O_2_ delivery to peripheral organs and tissues. Interestingly, TRPA1 acts as sensors for not only hypoxia but also for hyperoxia, suggesting that at least TRPA1 and other redox-sensitive TRP channels also transmit negative signals to suppress excessive O_2_ delivery responsible for harmful ROS production. These TRP channels may even contribute to a mechanism that maintains O_2_ availability of certain organs/tissues and their subareas at hypoxic levels compared with the atmospheric O_2_ level. It has indeed been reported that hypoxic levels are important in maintaining cellular conditions of certain types of cells in vivo [24, 75, 76, 98].

**Fig. 3:**
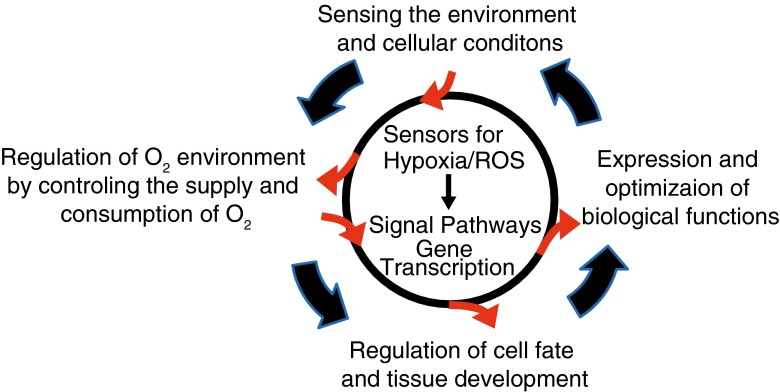
Concept of O_2_ remodeling. Hypoxia/ROS sensors detect deviation of O_2_ availability and transmit this information to neurons and/or humoral factors such as vascular endothelial growth factor to control O_2_ delivery. Furthermore, according to types, location, and cellular condition of tissues in the body, mitochondrial O_2_ consumption is regulated by mechanisms such as the Pasteur effect, which switches O_2_ dependence of ATP production

**Fig. 4:**
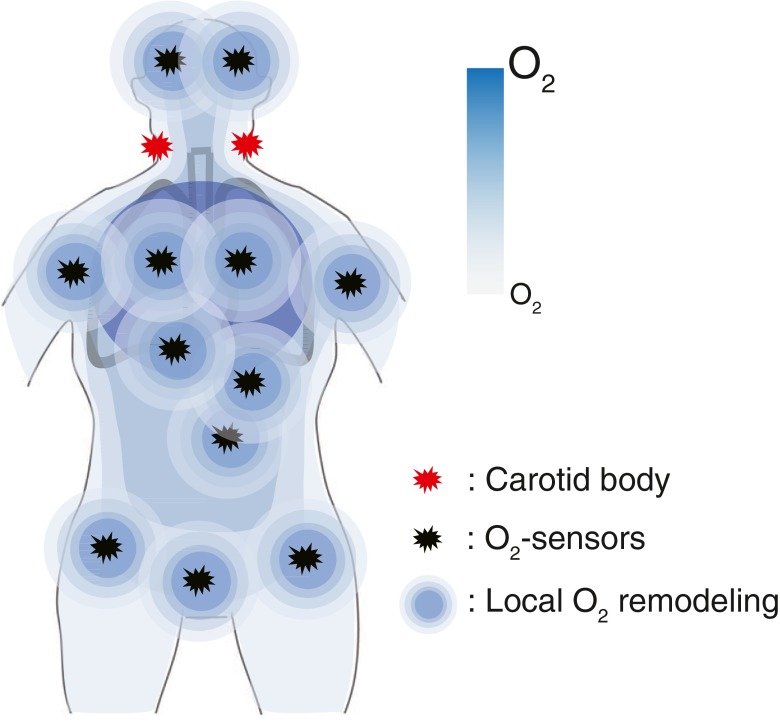
O_2_-sensitive receptors are localized ubiquitously present in a variety of tissues and organs. It is possible that TRP O_2_ sensors detect local O_2_ availability and contribute to fine tuning of local O_2_ levels, which cannot be accomplished by the carotid body alone, in the respective organs and tissues and in their subareas

The many lines of experimental evidence thus far have led us to propose the concept of “O_2_ remodeling” (Fig. 3). In O_2_ remodeling, O_2_-sensing chemoreceptors detect deviation of O_2_ availability and transmit this information to neurons and/or humoral factors, such as vascular endothelial growth factor [57, 93] to control O_2_ delivery. Also, according to the types, location, and condition (including O_2_ availability itself) of the tissues in the body, mitochondrial O_2_ consumption [88] is regulated by mechanisms such as the Pasteur effect, which switches O_2_ dependence of ATP production [109]. In the mechanism underlying O_2_ remodeling, O_2_ sensor TRP channels and redox-sensitive TRP channels play important roles, together with signaling cascades controlled by HIF/PHD [91, 112] and also by polysulfide redox factors [43]. Compared with the roles of HIF/PHD, those of TRP channels in controlling O_2_-triggered signaling cascades via signals of ions such as Ca^2+^ are still very elusive. As a readout of the signaling mechanism, O_2_ availability is adjusted to optimal levels, which enable sufficient cellular O_2_ supply for the activity and function of corresponding organs and tissues and at the same time, minimized production of excessive ROS and cellular damage. It is interesting to speculate that such “active” (not passive) optimization by O_2_ remodeling leads to the formation of a local O_2_ environment, in which population of cells behave as a unit for homeostasis that is responsible for the regulation of metabolism and development of organs and tissues in aerobic organisms, including as human beings (Fig. 4). We should note that TRPA1 and TRPM7 are not necessarily associated with control of O_2_ supply in all organs and tissues, considering their well-known ability to detect substances other than O_2_. However, we still consider it reasonable to expect modification by changes in O_2_ availability for cellular responses via these TRP channels activated by these other triggers.

## Conclusion

Identification of O_2_-sensing TRP channels opens a new area of oxygen physiology. In particular, wide tissue expression of O_2_-sensing TRPA1 and TRPM7 channels is indicative of “acute” O_2_-sensing capacity in diverse types of cells, tissues, and organs. This constitutes a considerable departure from the classical concept of respiratory physiology ascribing the powerful hypoxic chemoreflex solely to CB chemoreceptor excitation [81, 100]. In the case of TRPA1, hyperoxia-induced activation has been shown through the quantitative characterization of oxidation sensitivity of redox-sensitive TRP channels. The O_2_-sensing mechanisms involving TRPA1 and other oxidation-sensitive mechanisms may be important for maintaining O_2_ availability at certain hypoxic levels to avoid unnecessary and excessive production of ROS. In this review, we have suggested that “O_2_ remodeling,” in which cells comprising organs and tissues actively form a local in vivo O_2_ environment optimal for their function in the body, emerges as a new central concept for oxygen biology. This concept may allow us to systematically understand numerous physiological phenomena affected by O_2_ availability in aerobic organisms. In studying O_2_ remodeling, it is a tantalizing prospect to discover whether O_2_-sensing TRP channels are involved in the mechanisms underlying ‘chronic’ forms of hypoxic adaptation. Breakthrough studies on the time-dependent aspects (acute vs. chronic), as well as the concentration-dependent aspects (hypoxic vs. hyperoxic) of O_2_ remodeling would eventually result in a paradigm shift in our understanding of the biology of O_2_.
